# Demonstration of in-vivo simultaneous 3D imaging with ^18^F-FDG and Na^131^I using Compton–PET system

**DOI:** 10.1038/s41598-024-71750-3

**Published:** 2024-09-09

**Authors:** Donghwan Kim, Linlin Yan, Kenji Shimazoe, Hiroyuki Takahashi, Kenichiro Ogane, Masao Yoshino, Kei Kamada, Mizuki Uenomachi

**Affiliations:** 1 Department of Nuclear Engineering and Management, The University of Tokyo, 7-3-1 Hongo, Bunkyo City, Tokyo Japan; 2 Department of Bioengineering, The University of Tokyo, 7-3-1 Hongo, Bunkyo City, Tokyo Japan; 3 Department of Nuclear Medicine, International University of Health and Welfare, 1-4-3 Mita, Minato City, Tokyo Japan; 4 Institute for Materials Research, Tohoku University, 6-6-10 Aoba, Aramaki, Aoba-ku, Sendai, Miyagi 980-8579 Japan; 5 New Industry Creation Hatchery Center, Tohoku University, 6-6-10 Aoba, Aramaki, Aoba-ku, Sendai, Miyagi 980-8579 Japan; 6 Unit of Synergetic Studies for Space, Kyoto University, Kitashirakawa, Sakyo-ku, Kyoto, 606-8502 Japan

**Keywords:** PET, Compton camera, Thyroid cancer imaging, Imaging techniques, Molecular medicine, Cancer imaging

## Abstract

Simultaneous imaging of the SPECT tracer ^131^I and PET tracer ^18^F is important in the diagnosis of high- and low-grade thyroid cancers because high-grade thyroid cancers have high ^18^F-FDG and low ^131^I uptake, while low-grade thyroid cancers have high ^131^I and low ^18^F-FDG uptake. In this study, Na^131^I and ^18^F-FDG were simultaneously imaged using the Compton-PET system, in vivo. The angular resolution and sensitivity of the Compton camera with 356 keV gamma ray measured using a ^133^Ba point source were 12.3° and 2 × 10^−5^, respectively. The spatial resolution and sensitivity of PET were measured with a ^22^Na point source. The transaxial and axial spatial resolutions of the PET at the center of the FOV were 1.15 mm and 2.04 mm, respectively. Its sensitivity was 1.2 × 10^−4^. In-vivo images of the ^18^F and ^131^I isotopes were simultaneously acquired from mice. These showed that ^18^F-FDG was active in the heart, brown fat, and brain, while Na^131^I was active in the thyroid, stomach, and bladder. Artifacts were found in the Compton camera images when the activity of ^131^I was much lower than that of ^18^F. This study demonstrates the potential of simultaneous clinical imaging of ^18^F and ^131^I.

## Introduction

The in-vivo imaging of radioisotopes is significant for diagnosing various diseases in nuclear medicine. Single photon emission computed tomography (SPECT) and positron emission tomography (PET) are the most used methods^1,2^.

SPECT uses collimators, such as parallel holes and pin holes, made of materials that absorb gamma rays. A system response is based on the geometry of the collimators. SPECT can perform multiple nuclide imaging by accepting different energy windows corresponding to different gamma ray energies; however, it is limited to low-energy gamma rays because high-energy gamma rays can penetrate the collimators. The penetrating gamma rays generate a background in the image, degrading the correlation between the actual activity and voxel intensity in the image. It indicates the possibility that the Compton camera, which will be explained later, might outperform SPECT if the incident energy is high^3^.

PET takes gamma rays from positron–electron annihilations. The gamma rays are emitted collinearly. The energy of both gamma rays is 511 keV. If the two annihilation gamma rays interact with the detectors within a short time, a coincidence event is recorded. The image is reconstructed with lines of response (LORs) using the collinear property of the direction of the annihilation gamma rays. All positron–electron annihilation events generate gamma rays of the same energy. Therefore, identifying different isotopes that emit positrons using only coincidence events of two 511 keV gamma rays is impossible. Prompt gamma rays can be used to identify different positron emitters. Several studies have been conducted on multinuclide imaging of PET tracers using prompt gamma rays^4–6^. In these studies, extra detectors were used to increase the sensitivity of triple coincidence, two annihilation gamma rays, and one prompt gamma ray.

The Compton camera is an imaging device that captures images of single photon-emitting nuclides. Unlike SPECT, Compton camera captures coincidence events with Compton scattering and the absorption of scattered gamma rays. It also collects energy information from events. The position information of the scatter and absorption and the energy deposit information in the scatter are used to generate a cone which is the system response of the Compton camera. Half the apex angle of the Compton cone is calculated as

1 $$\theta =\text{acos}\left(1-{m}_{0}{c}^{2}\left(\frac{1}{{E}_{inc}-{E}_{sca}}-\frac{1}{{E}_{inc}}\right)\right)$$

where ${m}_{0}{c}^{2}$ is the mass of electron at rest, ${E}_{inc}$ is the energy of the incident gamma ray, and ${E}_{sca}$ is the deposit energy of the scatter detector. Compton cameras have been used in various fields, including space telescopes, environmental monitoring, beam-range monitoring during particle therapy, and preclinical and clinical imaging applications^7–11^.

Another advantage of the Compton camera is that it can be more easily combined with PET than with SPECT because it does not require collimators. Several studies have been conducted on multinuclide imaging using a Compton camera and PET^9,12–15^. Combining PET and Compton cameras as SPECT scanners for simultaneously imaging PET and SPECT tracers has many advantages. This relieves the burden on patients who require multiple PET and SPECT scans. If multiple tracers are imaged separately, the patient’s physiological conditions may vary and affect the images, reducing the accuracy of the evaluation. Separate PET and SPECT scans require separate registrations. In addition, patients receive a higher dose from computed tomography (CT) scans, approximately 5–9 mSv per scan, because PET and SPECT require CT images for attenuation correction. Simultaneous measurement can facilitate the diagnosis of tumors with different uptake values with different tracers, such as differentiated and undifferentiated thyroid carcinoma^16^.

Previously, we demonstrated the world’s first Compton-PET, in which two Compton cameras were used to image SPECT and PET tracers^17^. Subsequently, we demonstrated the world’s first Compton–PET simultaneous in-vivo imaging of ^111^In and ^18^F, followed by the simultaneous 2D imaging of ^131^I and ^18^F using Compton–PET with two Compton camera modules facing each other^13,18^. This study demonstrates simultaneous in-vivo 3D imaging of Na^131^I and ^18^F-FDG using a ring Compton–PET system comprising eight Compton camera modules with crystal-by-crystal parallel and independent data acquisition.

Simultaneous imaging of Na^131^I and ^18^F-FDG is significant for thyroid cancer therapy. In general, high-grade thyroid cancers, such as undifferentiated carcinomas, have high ^18^F-FDG uptake and poor iodine uptake. On the other hand, low-grade thyroid cancers such as papillary carcinoma have low ^18^F-FDG uptake and high iodine uptake. In high-grade undifferentiated thyroid carcinomas, iodine uptake is poor; thus, internal ^131^I therapy is not applicable. Low-grade thyroid cancers, such as papillary thyroid cancer, are treated using ^131^I therapy. Thyroid scintigraphy may not be able to perceive iodine uptake during thyroid cancer therapy with ^131^I. In such cases, there are two possibilities. Either the treatment has been successful and the thyroid tissue has been completely treated or malignant transformation of the thyroid cancer to undifferentiated carcinoma has occurred. In the case of malignant transformation of thyroid cancer to undifferentiated carcinoma, ^18^F-FDG PET is recommended to assess the primary tumor and metastases. However, it is not always performed due to the heavy burden on patients and healthcare providers as it is performed in a different modality. Simultaneous ^18^F-FDG PET imaging at the end of ^131^I thyroid therapy could potentially detect malignant transformation of undifferentiated carcinoma having poor prognosis at an early stage.

The system's performance was evaluated at 356 and 511 keV using a Compton camera and PET on the sensitivity and angular resolution with ^133^Ba and ^22^Na point sources. Five-rod phantom images of ^18^F-FDG and Na^131^I were taken.

## Results

### Point-source measurement

**Fig. 1:**
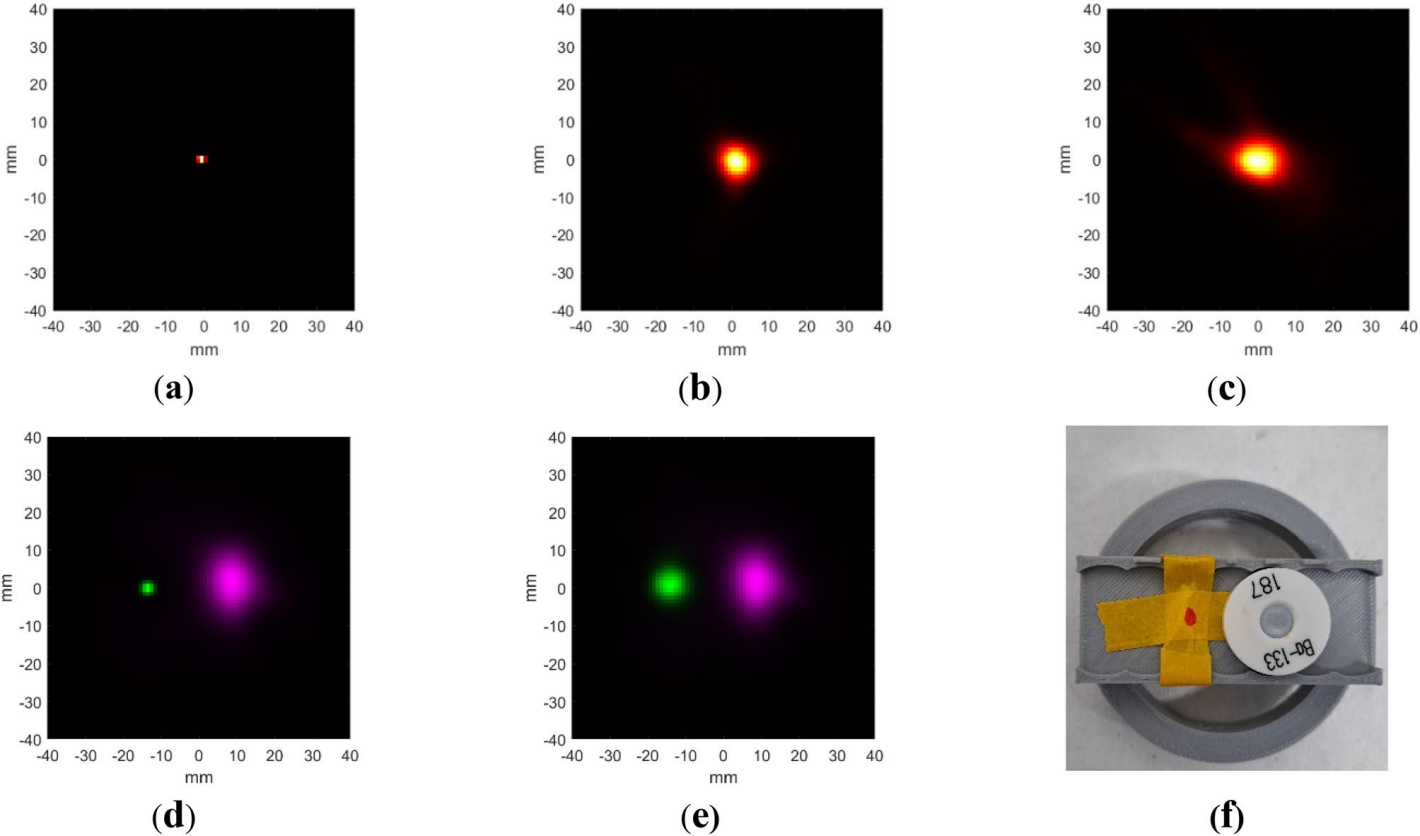
Images from point-source measurements. (**a**) Na-22 PET, (**b**) 511 keV Compton camera, (**c**) 356 keV Compton camera, (**d**) PET (green) and 356 keV Compton camera (magenta), (**e**) 511 keV (green) and 356 keV (magenta) Compton camera, (**f**) The picture of the geometry of ^22^Na and ^133^Ba simultaneous imaging. The red point is the position of the ^22^Na source.

Figure 1a–c shows reconstructed images of the ^133^Ba and ^22^Na point sources at the center of the field of view (FOV). The activities of ^22^Na and ^133^Ba were 0.1 MBq and 0.5 MBq, respectively. These were successfully localized. The images were reconstructed using five iterations of list-mode ordered subset expectation maximization (LMOSEM) for the Compton camera and 10 iterations of maximum likelihood expectation maximization (MLEM) for PET.

Figure 1d, e shows the simultaneous reconstruction of the point sources, and Fig. 1f shows the source geometry when the images shown in Fig. 1d,e were captured. They were reconstructed separately and fused after the reconstruction. Compton camera images were obtained from five iterations of LMOSEM. The PET image was the result of 10 MLEM iterations. They show a separation of approximately 20 mm which is the same as the actual value.

**Fig. 2:**
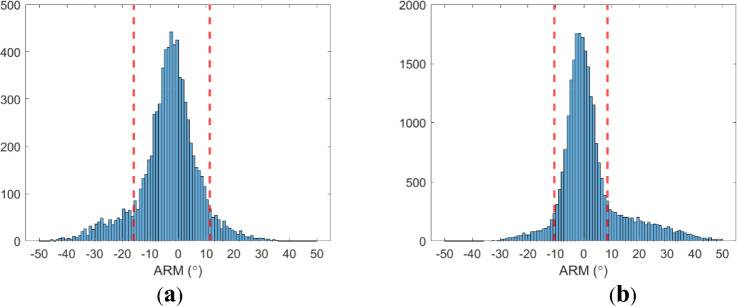
ARM measurement plots. (**a**) 356 keV, (**b**) 511 keV. The red dashed lines indicate the region used for sensitivity calculation.

The sensitivity and angular resolution measure (ARM) were determined from point-source measurements of ^133^Ba and ^22^Na at the center of the FOV. The full width at half maximum (FWHM) of ARM of the Compton camera at 356 keV and 511 keV were 12.3° and 9.49°, respectively. The transaxial and axial spatial resolutions of PET at the center of the FOV were 1.15 mm and 2.04 mm, respectively. Figure 2 shows the ARM distribution and 2 × FWHM of the ARM region for each energy level.

The sensitivities of the Compton camera at 364 and 511 keV were 2.0 × 10^−5^ and 6.3 × 10^−5^. The sensitivity of PET was 1.2 × 10^−4^. The Compton camera sensitivity at 511 keV was significantly higher than that at 364 keV. This was because two 511 keV gamma rays were emitted from one positron, and the 511 keV scatter energy window was larger.

### Phantom imaging

**Fig. 3:**
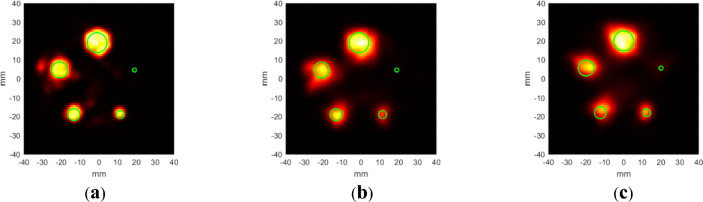
Image reconstruction results from the 3D-printed phantom. The green circles indicate 70% of the original diameter and the locations. (**a**) PET, MLEM 30 iterations, (**b**) Compton camera at 511 keV, OSEM 20 iterations, (**c**) Compton camera at 364 keV, OSEM 20 iterations.

Figure 3 shows the results of the phantom imaging using the Compton camera and PET. The Compton camera and PET images at 511 keV showed that each rod was visible, except for the 3 mm rod. The exact reason for the 3 mm rods not being visible at 511 keV is not clear. Considering that PET exhibits better spatial resolution than the 364 keV Compton camera and the 364 keV Compton camera shows the 3 mm rod with low intensity, ^18^F-FDG was probably not properly injected into the 3 mm rod when making the phantom. The solid green circles show the expected relative positions of the sources compared with the position of the 15 mm diameter circle. The size of the circles is 70% of the actual size. The PET and Compton camera images at 511 keV show almost no offset. By contrast, the Compton camera image at 364 keV shows a slight offset from the expected positions, although it is not completely out of the region. The color range corresponds to the full dynamic range of each image.

### In-vivo imaging of mice

**Fig. 4:**
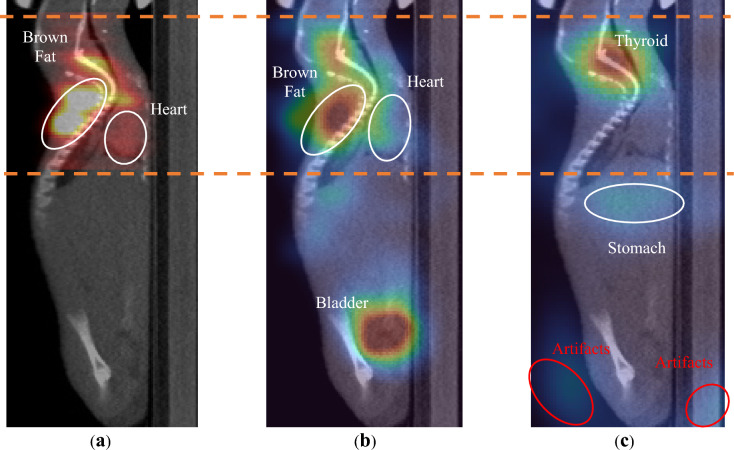
Image Reconstruction of mouse 1 overlayed on CT images. (**a**) PET image. (**b**) Compton camera image at 511 keV. (**c**) Compton camera image at 364 keV. The orange dashed lines indicate the FOV of PET.

Figure 4 shows the reconstructed PET images and Compton camera images of Mouse 1 at 511 keV and 364 keV. The PET images were obtained from 20 MLEM iterations and scaled to 50% of the maximum intensity. The Compton camera images were obtained from five OSEM iterations and scaled to 70% of the maximum intensity. The PET and Compton camera images were obtained from the same slices. The PET images showed a limited FOV, because they could not be imaged outside the detector size. By contrast, the Compton camera can be used to visualize outside the axial FOV of PET. The CT images were taken separately from the Clairvivo CT (Shimadzu, Japan). The registration of CT and functional images is not precise. The regions of interest (ROIs) were taken based on functional images. ^18^F accumulated in the brown fat and bladder. ^131^I accumulated primarily in the thyroid glands.

However, artifacts were observed in the 364 keV Compton camera image because of the low activity of ^131^I compared with ^18^F, as shown in Fig. 1 in the supplementary material. The artifacts were caused by two Compton scatter events at 511 keV from ^18^F, which left a trace that satisfied the 364 keV energy windows in Table 2.

**Fig. 5:**
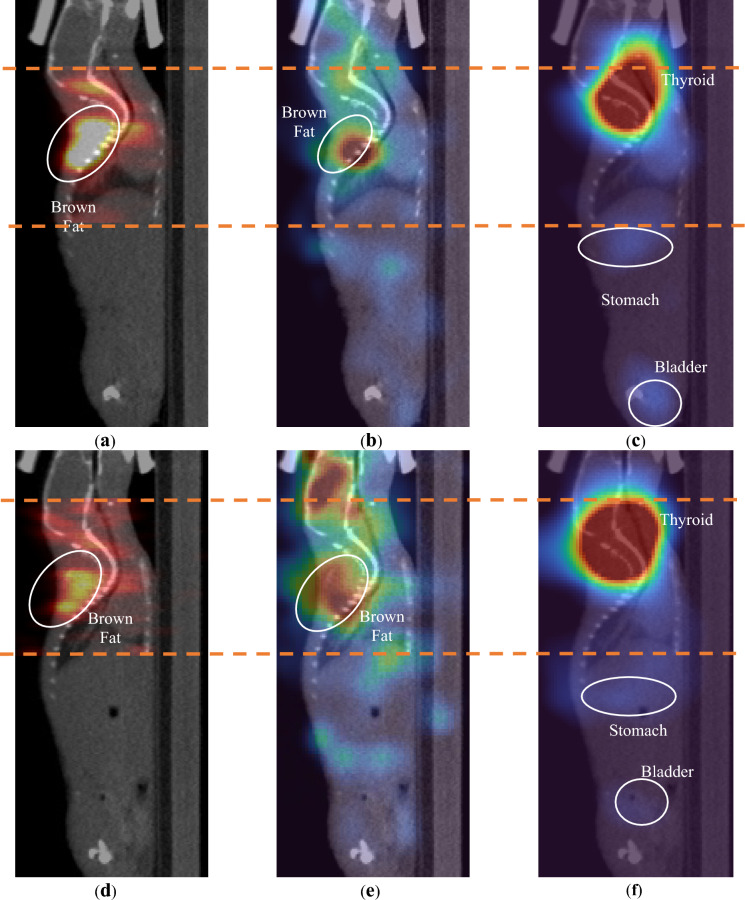
Reconstructed images of mice 2 and 3 overlayed on CT images. (**a**) PET image of mouse 2. (**b**) Compton camera image of mouse 2 at 511 keV. (**c**) Compton camera image of mouse 2 at 362 keV. (**d**) PET image of mouse 3. (**e**) Compton camera image of mouse 3 at 511 keV. (**f**) Compton camera image of mouse 3 at 362 keV.

Figure 5 shows the reconstructed images of Mouse 2 and Mouse 3. PET and Compton camera images were obtained from the same slices. The PET and Compton camera images at 511 keV were obtained under the same conditions as those in Fig. 4. However, the Compton camera image at 364 keV was scaled to 30% of the maximum to visualize the stomach areas exhibiting relatively less accumulation than those of Mouse 1. For both mice, the images did not exhibit significant artifacts at 364 keV owing to their higher relative activity than ^18^F. Mouse 2 exhibited a slightly higher FDG concentration in the brain than Mouse 1 and Mouse 3. Mouse 2 and Mouse 3 did not show high accumulation in the bladder. This indicates that the Compton camera could effectively capture the different physiologies of the mice.

**Fig. 6:**
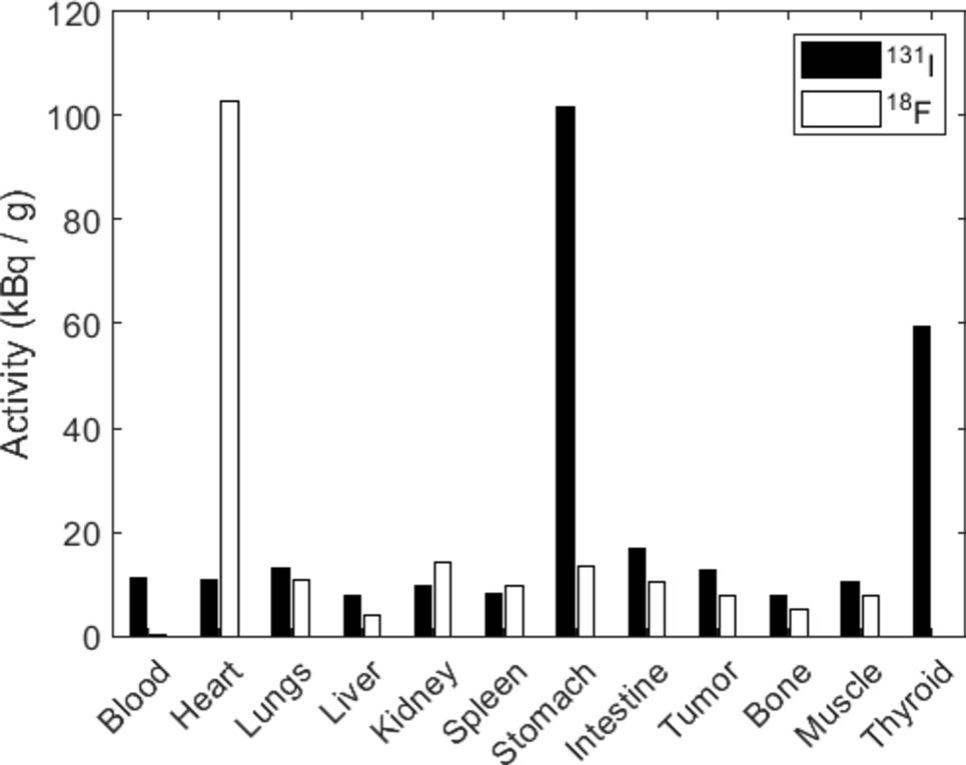
^131^I and ^18^F activity of organs from mice 4 and 5 measured by an auto-well gamma counter.

Figure 6 shows the biodistributions in Mouse 4 and Mouse 5. The organs where radioactivity was expected were chosen and the radioactivity was measured using an auto-well gamma counter. The heart contained the most ^18^F-FDG, whereas the stomach and thyroid contained the most Na^131^I. The two organs with the highest iodine accumulation matched the results of the iodine images, although the highest was in the thyroid according to the image. The ^18^F accumulation in brown fat was not measured; therefore, it could not provide the relative activity between the heart and brown fat. Tumors were planted in all the mice. However, no tumors were well visualized owing to low communication, as shown in Fig. 6.

## Discussion

We conducted a performance evaluation of Compton-PET, which features crystal by crystal parallel and independent readouts, and in-vivo imaging of mice using gamma ray energies at 356 keV and 511 keV. A combination of these energies is significant for thyroid cancer imaging using ^18^F-FDG and Na^131^I. Thyroid cancer may malignantly transform into undifferentiated carcinomas where there is no uptake of iodine. However, glucose uptake is enhanced in undifferentiated carcinomas. Thus, ^131^I scintigraphy alone cannot be used to treat or identify malignant transformation. Therefore, post-treatment blood examinations in routine clinical practice to determine the effectiveness of treatment cannot guarantee the presence of undifferentiated carcinomas.

The spatial resolution performance of the Compton-PET system was evaluated at 511 keV and 356 keV, corresponding to 7.7 mm and 10 mm spatial resolution at the center of the FOV according to Eq. (3). This spatial resolution was poor compared to that of preclinical multi-pinhole SPECT systems^19,20^. However, the Compton camera has a larger axial FOV and less weight than multi-pinhole SPECT systems. The weight of the multi-pinhole SPECT system is heavy due to the collimators and a large area of detectors needed for axial FOV similar to that of Compton cameras. Therefore, the Compton-PET or an equivalent system is a lighter and cheaper solution to simultaneous SPECT and PET imaging.

The performance of the Compton camera is expected to be poor compared to that of the clinical SPECT in terms of spatial resolution. The distance from the center of the FOV to the surface of the camera is approximately 200 mm in clinical SPECT. The spatial resolution of the current Compton camera at the center of the FOV is approximately 44 mm. This is four times greater than the resolution of the clinical SPECT which is approximately 10 mm^21^. Further studies will be conducted on clinical applications, including using the laparoscopic approach^22^.

The angular resolution of the Compton camera depends on the energy and spatial resolution of the detectors^23,24^. Using semiconductor detectors, such as silicon, cadmium telluride (CdTe), cadmium zinc telluride (CZT), thallium (I) bromide (TIBr), and high purity germanium (HPGe) can provide better angular resolution because they have better energy resolutions^12,25–29^. The point spread function (PSF)-MLEM can also enhance spatial resolution^30^. With these improvements, the Compton-PET system has the potential to be utilized in clinical imaging for thyroid cancer imaging.

**Fig. 7:**
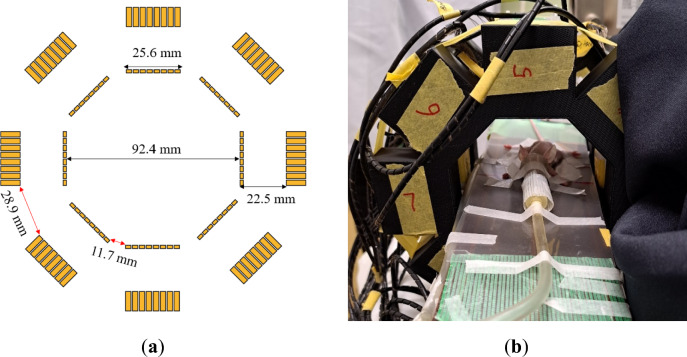
(**a**) Schematic of the Compton-PET system. Red arrows indicate the gap between scatterers and absorbers. (**b**) The Compton-PET system with a mouse for measurements.

The sensitivities were low owing to the geometry of the system. Figure 7a shows a schematic of the Compton-PET system. A large amount of radiation escaped from the system through the gaps between detectors. The absorber gap was larger than the scatterer gap. It can accommodate additional detectors within the absorber gap. Covering the entire cylinder with absorbers will significantly increase the sensitivity. Filling the gap will also improve the quality of PET images^31^. Therefore, reducing the gap between detectors will significantly enhance the performance of Compton-PET. In high activity conditions, the crystal-by-crystal parallel and independent readout of the Compton-PET approach can have high efficiency because only one crystal is paralyzed when the crystal interacts with radiation. In case the charge division method is used, which is typically adopted, the whole block is paralyzed when one crystal interacts with radiation.

The relatively higher activity of ^18^F than ^131^I caused artifacts in the 364 keV Compton camera image that were not observed when the activity of ^131^I was high. This implies that there might be a deviation in the linearity between the observed image intensity and the actual activity. For this approach to be quantitatively precise, a method for the deviation should be investigated. A simple strategy is to administer a higher activity of lower energy sources (^131^I in this study).

Currently, this system comprises a single ring. As observed in the PET images, the axial FOV was limited to 25.6 mm, which was short for the entire mouse body. Consecutive measurements at different axial positions can be performed for a whole-body PET in the mice, but increasing the number of rings will ease the measurement procedures and improve the sensitivity of the system.

This study performed in-vivo imaging of mice and performance evaluation of the Compton-PET system with the target isotopes of ^131^I and ^18^F, which are important for thyroid cancer therapy and imaging. The Compton-PET system successfully demonstrated simultaneous ^131^I and ^18^F imaging with mice. Although the Compton-PET system has advantages over previously developed simultaneous PET-SPECT tracer imagers using collimators, such as larger FOV and lighter weight, the poor angular resolution of the Compton camera is the most significant challenge for clinical application. Studies will be conducted in the future to improve the system by replacing scatterers for better angular resolution, adding extra absorbers, and creating more rings for larger FOV for PET and higher efficiency.

## Materials and methods

### Compton–PET system

This study used the Compton–PET system comprising Ce:Gd_3_Al_2_Ga_3_O_12_ (HR-GAGG) scintillation crystals^32^. Figure 7 shows the schematics and the actual system with a mouse. It comprised eight Compton camera modules. Each Compton camera module comprised a scatterer and an absorber. Eight Compton camera modules were assembled to create an octagonal ring structure. A one-ring structure was used in this study. The scatterer and absorber had $8\times 8$ crystal arrays. The size of the scatterer and absorber crystals were $2.5\;{\text{mm}} \times 2.5\;{\text{mm}} \times 1.5\;{\text{mm}}$ and $2.5\;{\text{mm}} \times 2.5\;{\text{mm}} \times 9\;{\text{mm}}$, respectively. The pitch between the crystal elements was 3.2 mm. Each crystal was coupled to one pixel of a Hamamatsu MPPC S13361-3050 with a 3.2 mm pitch. The distance between the surface of the scatterer and the absorber was 22.5 mm. The distance between the surfaces of the scatterers that are opposite each other was 46.2 mm.

### Data acquisition system

The signals from each MPPC pixel were processed independently and in parallel. The pulse signal was processed using the dynamic time-over-threshold (dTOT) method^33^. The digital outputs from the MPPC pixels and dTOT circuit pair were counted, synchronized, and transferred to a PC with a PETnet system^34^. The clock speed of PETnet for the timing and TOT measurements was 125 ps.

### Image reconstruction

3D-MLEM was used for the PET image reconstruction with a multi-ray approach for the system matrix^35,36^. 3D-LMOSEM with eight subsets was used to reconstruct the Compton camera image^37^.

A voxel-based system model calculation was used for the Compton camera to reflect the angular uncertainty of the energy resolution of the detectors^24,38^.

### Mice preparation

Three mice were used for imaging, and two were used for the biodistribution study. The mice were administered with Na^131^I and ^18^F-FDG. One isotope was administered to each mouse for the biodistribution studies. Na^131^I was orally administered 19 h before imaging. ^18^F-FDG was administered through the tail vein 1 h before imaging. Each mouse was scanned for 30 min. This experiment was approved by the Committee of the University of Tokyo and was performed in accordance with institutional guidelines and regulations. The experiment protocol complied with the ARRIVE guidelines. After the experiments, the mice were anesthetized with isoflurane and then euthanized by cervical dislocation.

Table 1 lists the activities administered to each mouse. Some of the administered Na^131^I to Mouse 1 was lost because Mouse 1 vomited after administration. Therefore, the actual dose was lower than the initially administered dose. The activity of administered ^131^I was less than 1.52 MBq because the mouse vomited some of it, and the loss could not be measured.

**Table 1:** Levels of activities of ^18^F and ^131^I on each mouse.

	^18^F-FDG activity (MBq)	Na^131^I activity (MBq)
Mouse 1	0.58	Less than 1.52
Mouse 2	0.33	2.13
Mouse 3	0.44	2.74
Mouse 4	None	2.44
Mouse 5	0.45	None

### Point-source measurement

The point sources of ^133^Ba and ^22^Na were measured to evaluate the FWHM of the ARM at 356 keV and 511 keV. ARM is defined as

2 $$ARM={\theta }_{G}-{\theta }_{E},$$

where ${\theta }_{G}$ is the scattering angle calculated from the geometric projection, and ${\theta }_{E}$ is the scattering angle calculated from the energy deposits. For the ARM measurements, the sources were placed at the center of the FOV. The sensitivity was calculated from the point sources at the center of the FOV. It was calculated as the total number of recorded events with $2\times$ FWHM of the ARM divided by the total number of disintegrations. The FWHM of the ARM was measured using Lorentzian fitting. The images were reconstructed simultaneously by placing the sources 20 mm apart.

The sensitivity of the Compton camera to each energy level was measured based on the FWHM of the ARM. It was assumed that coincident events that were far away from the peak of the ARM distribution did not provide meaningful information for image reconstruction. The ROI for the sensitivity calculation was set $2\times$ FWHM of the ARM. Sensitivity is the number of events within the ROI divided by the number of disintegrations.

PET sensitivity was measured by dividing the number of coincidence events by the number of disintegrations.

The following equation was used in the calculation of spatial resolution at the center of the FOV:

3 $$FWHM \left(\text{mm}\right)=l\times \text{tan}\left(FWHM of ARM\right)$$

where $l$ is the distance between the scatterer surface and the point of interest. The spatial resolution at the center of FOV was evaluated with 10 iterations of MLEM.

### Phantom measurement

**Fig. 8:**
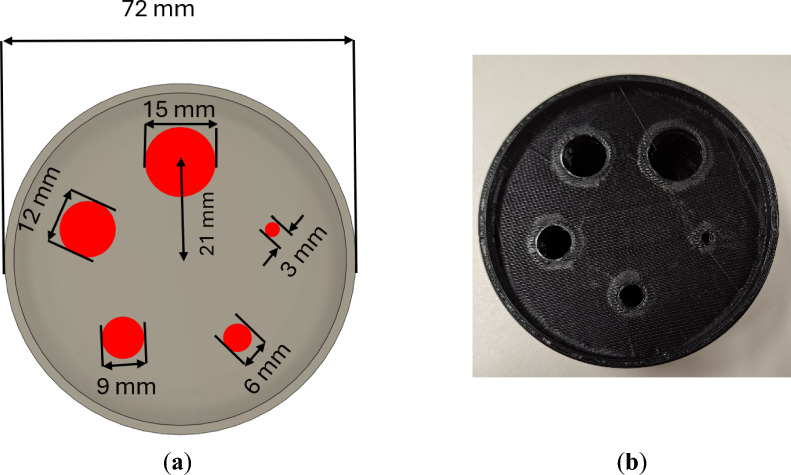
(**a**) The schematic of the phantom used in this study. (**b**) A photograph of the phantom printed from a 3D printer.

This study used a 3D-printed phantom. The structure comprised five rods of 15 mm, 12 mm, 9 mm, 6 mm, and 3 mm diameters. The distance from the transaxial center to the center of each circle was 21 mm. Figure 8 shows the phantoms used in this study. The heights of all the rods were set to 5 mm. The activity densities of ^18^F-FDG and Na^131^I measurements were 1.68 MBq/mL and 1.151 MBq/mL, respectively. The phantom measurement for both isotopes was 1 h.

### Data acquisition

This study independently processed the coincidence data for Compton imaging and PET. Time windows of 250 ns and 80 ns were used for Compton imaging and PET, respectively. Table 2 lists the energy windows of the Compton camera. ^133^Ba had a higher low threshold for scatter energy owing to the characteristic X-rays from ^133^Ba and ^137^Cs (32 keV and 31 keV, respectively). An energy window of 459.9 keV < *E*_*abs*_ < 562.1 keV was for PET data acquisition.

**Table 2:** Energy windows for data acquisition of Compton camera of each incident gamma energy.

Incident energy (keV)	Scatter energy window	Sum energy window
364 (^131^I)	30 keV < *E*_*sca*_ < 70 keV	327.6 keV < *E*_*sca*_ + *E*_*abs*_ < 400.4 keV
356 (^133^Ba)	45 keV < *E*_*sca*_ < 70 keV	320.4 keV < *E*_*sca*_ + *E*_*abs*_ < 391.6 keV
511 (^18^F)	330 keV < *E*_*sca*_ < 120 keV	459.9 keV < *E*_*sca*_ + *E*_*abs*_ < 562.1 keV

## Supplementary Information

Supplementary Figures.

## Data Availability

The datasets used and/or analyzed during the current study are available from the corresponding author upon reasonable request.
