# Supplementary material for: Demonstration of in-vivo simultaneous 3D imaging with 18F-FDG and Na131I using Compton–PET system
Source: Sci Rep. 2024 Sep 9;14:20946. doi: 10.1038/s41598-024-71750-3 (PMC11385225; doi:10.1038/s41598-024-71750-3)
Supplement: Supplementary file 1 — Supplementary Figures. [file 41598_2024_71750_MOESM1_ESM.pdf]

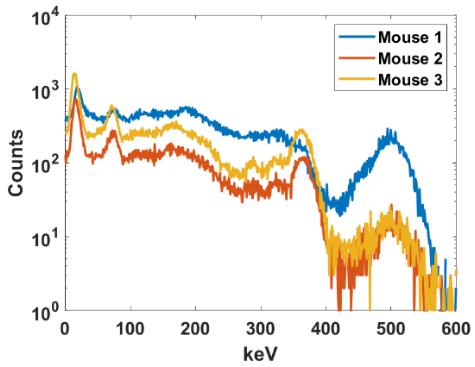

Fig. 1. Energy spectra of the three mice measured in this study. Mouse 2 and Mouse 3 show distinctive peaks at 364 keV, and all mice show clear peaks at 511 keV.

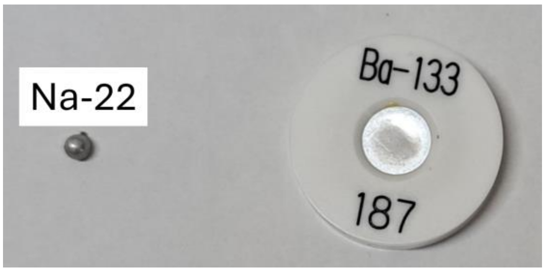

Fig. 2. A photograph of the point sources used in this study.
